# Supplementary figures and images for: Redox regulation and dynamic control of brain-selective kinases BRSK1/2 in the AMPK family through cysteine-based mechanisms
Source: eLife. 2025 Apr 2;13:RP92536. doi: 10.7554/eLife.92536 (PMC11964447; doi:10.7554/eLife.92536)

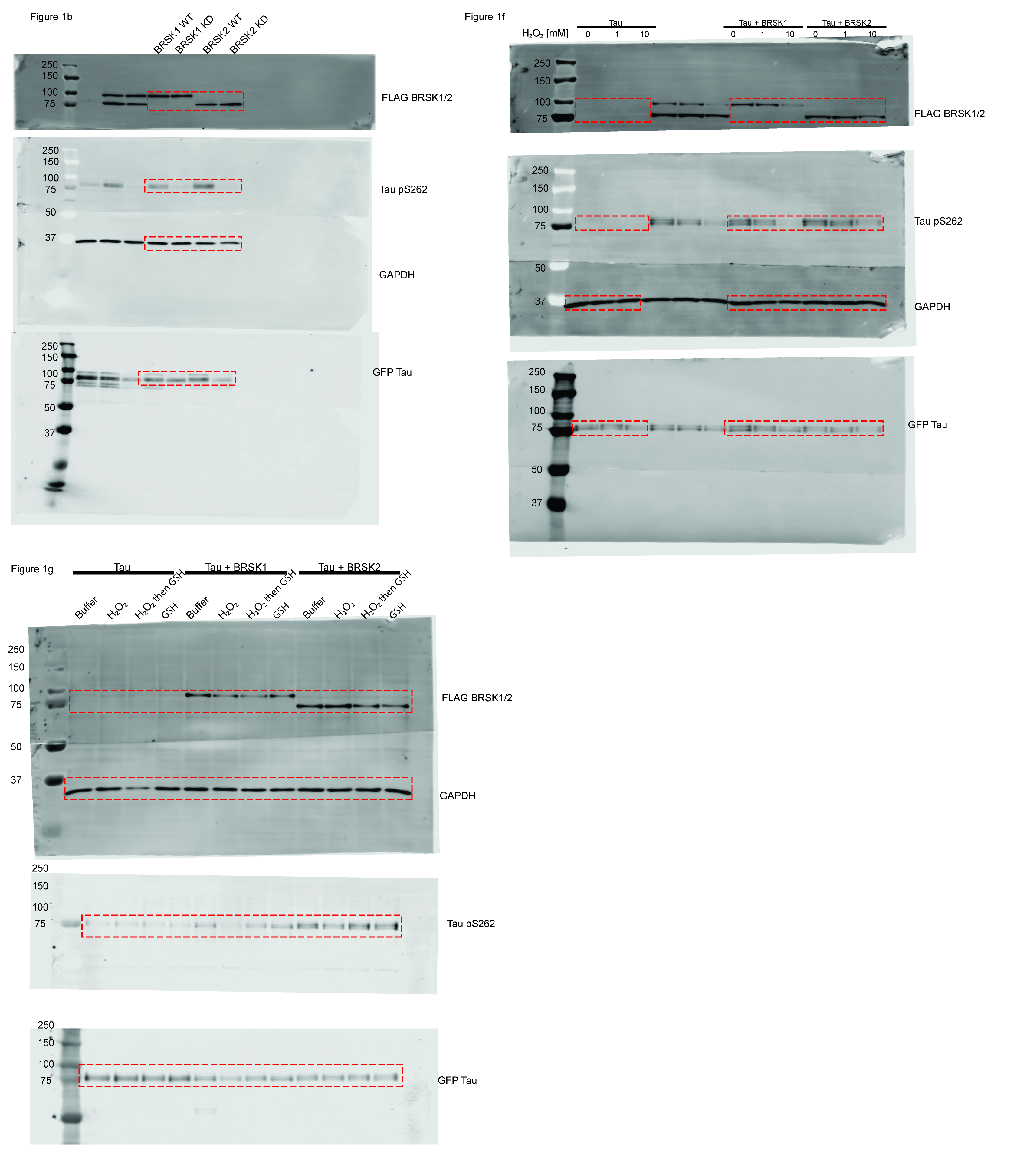

Supplement: Figure 1—source data 1. [file elife-92536-fig1-data1.zip › Figure 1-source data/Figure 1 - source data.tif]

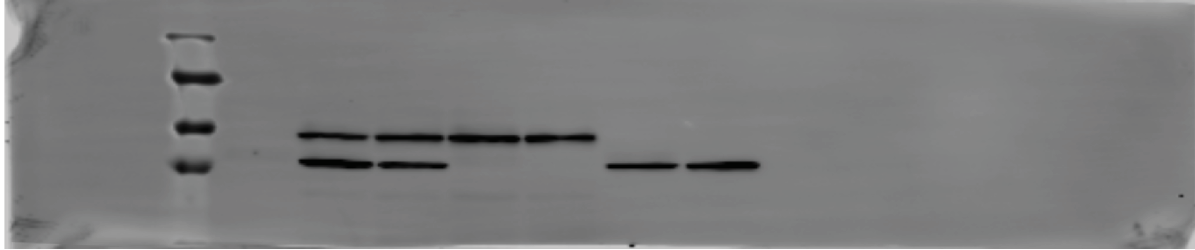

Supplement: Figure 1—source data 2. [file elife-92536-fig1-data2.zip › Figure 1-original source data/Figure 1 - source data 1b/1b_BRSK1_BRSK2.tiff]

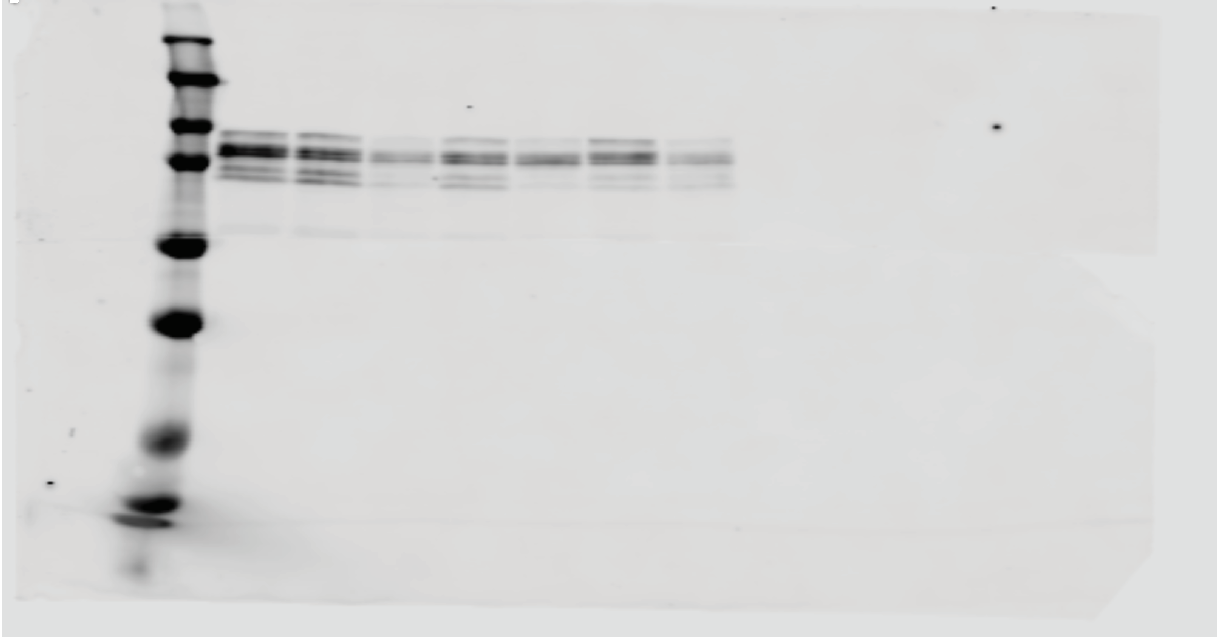

Supplement: Figure 1—source data 2. [file elife-92536-fig1-data2.zip › Figure 1-original source data/Figure 1 - source data 1b/1b_TAU.tiff]

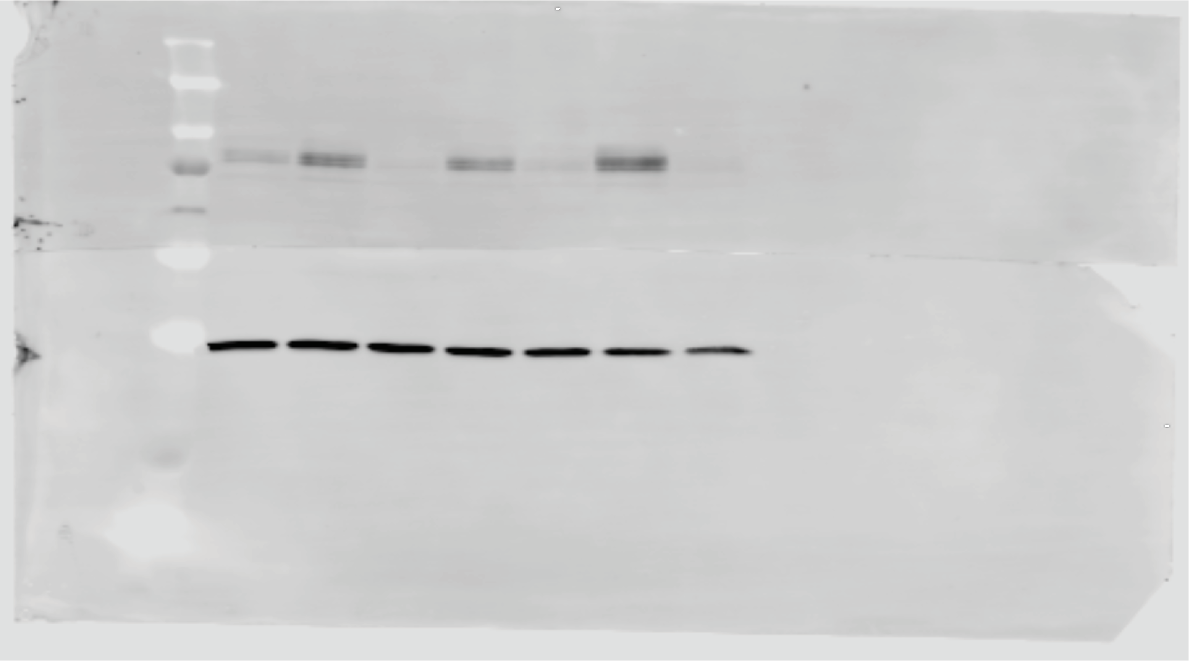

Supplement: Figure 1—source data 2. [file elife-92536-fig1-data2.zip › Figure 1-original source data/Figure 1 - source data 1b/1b_TAU_pS262_GAPDH.tiff]

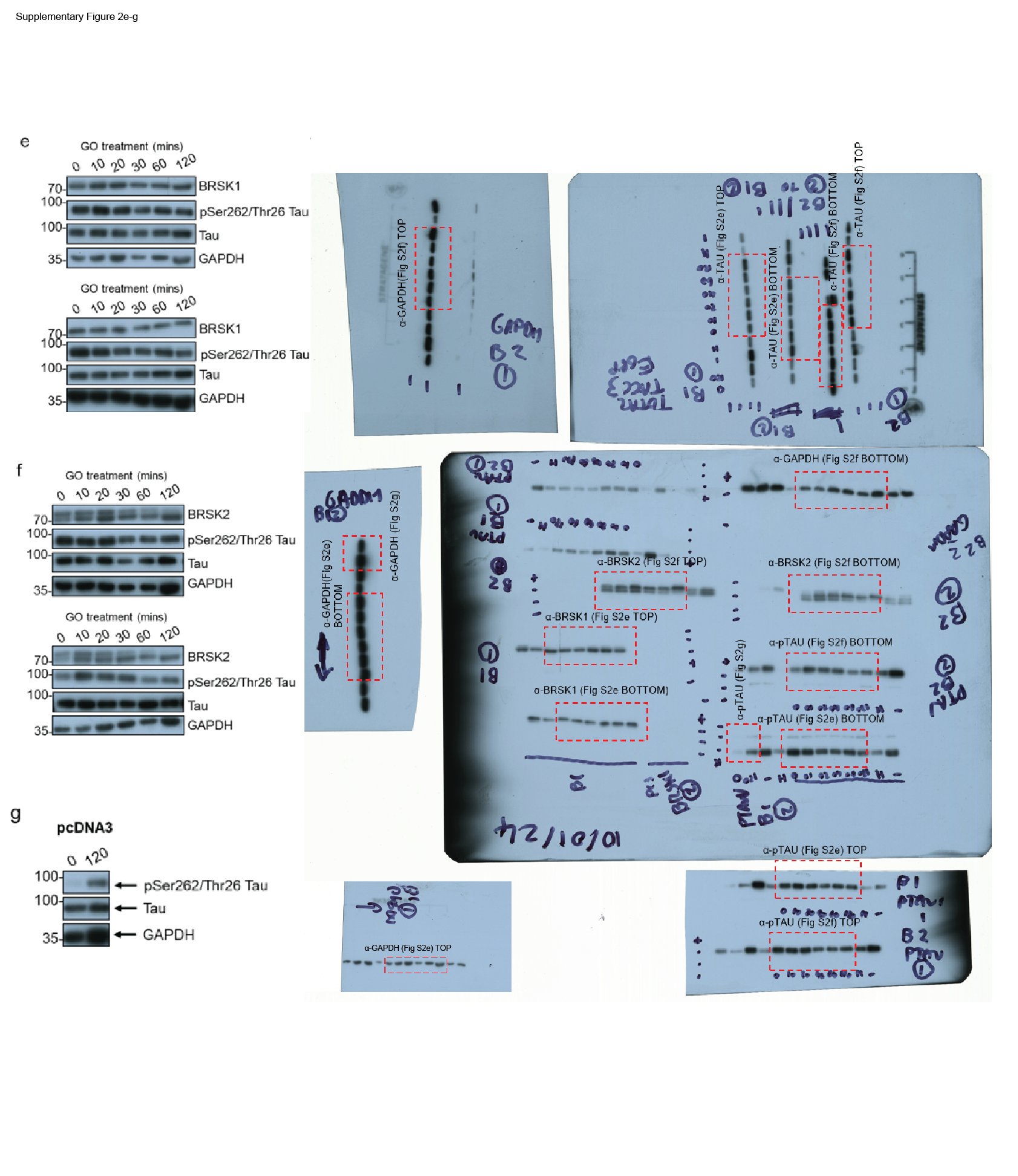

Supplement: Figure 1—figure supplement 1—source data 1. [file elife-92536-fig1-figsupp1-data1.zip › Figure 1 - figure supp 1 Source Data/Figure 1 - figure supp 1.png]

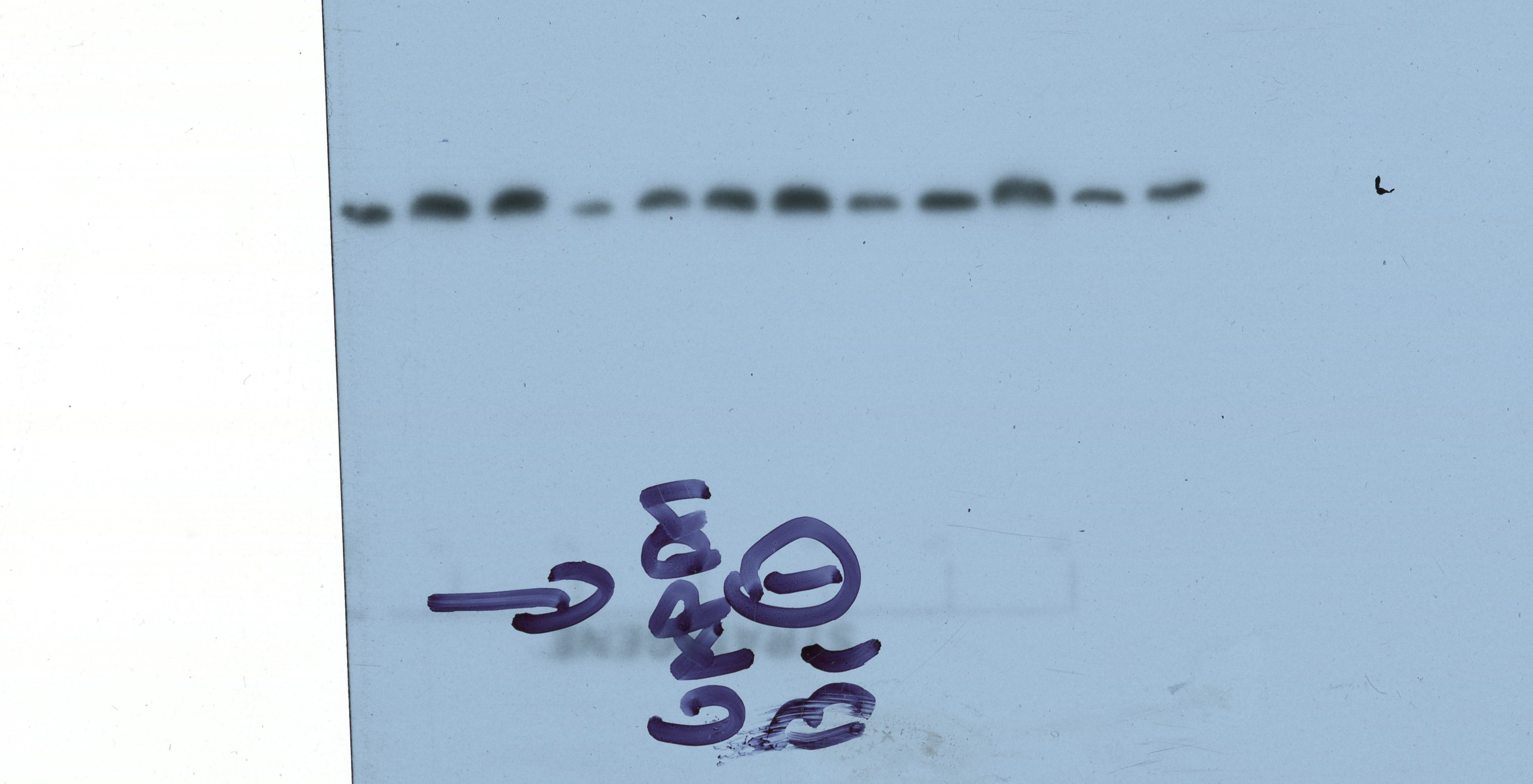

Supplement: Figure 1—figure supplement 1—source data 2. [file elife-92536-fig1-figsupp1-data2.zip › Figure 1 figure supp 1-original source data/Figure 1 figure supp 1-original source data GAPDH BRSK1.jpg]

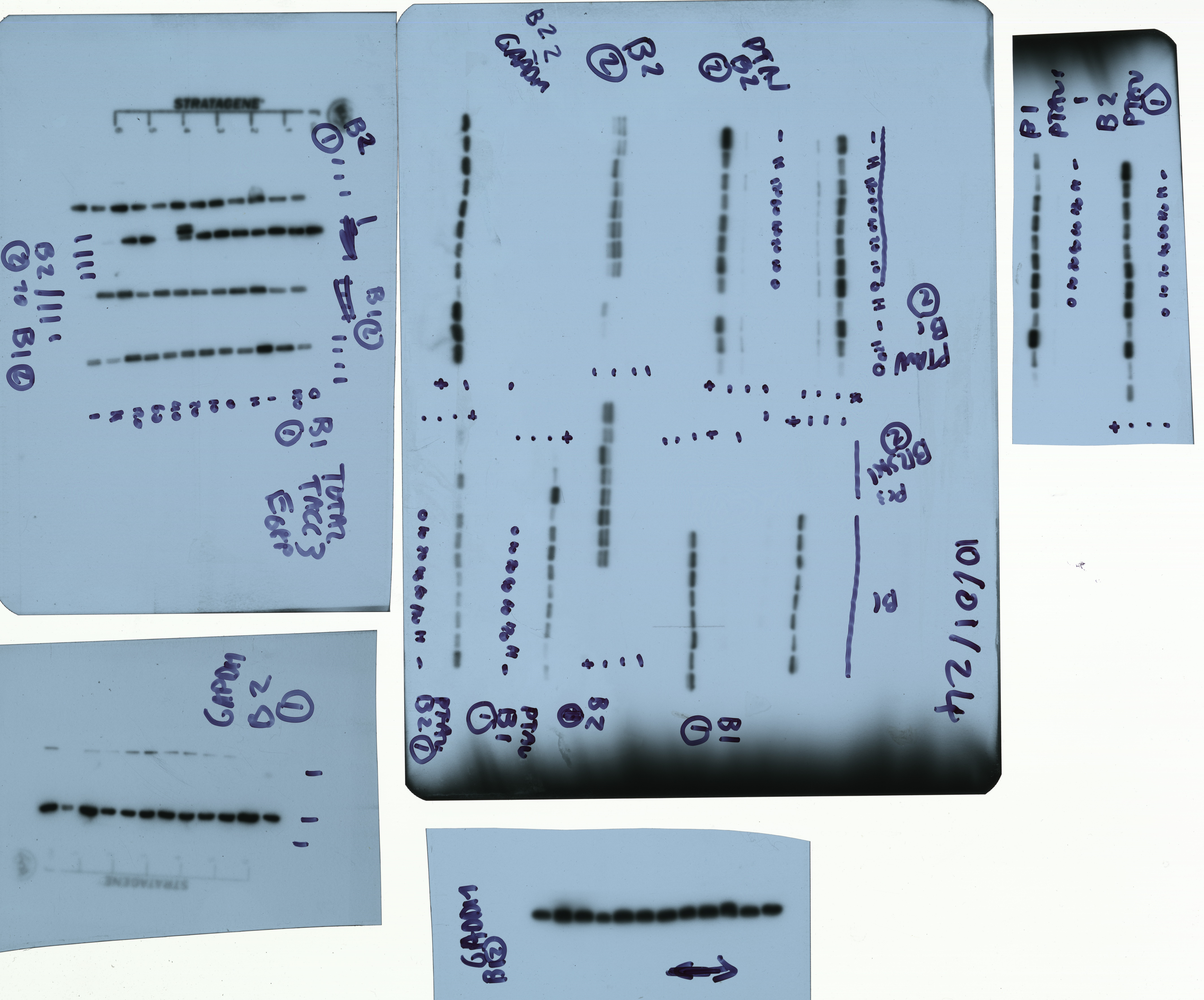

Supplement: Figure 1—figure supplement 1—source data 2. [file elife-92536-fig1-figsupp1-data2.zip › Figure 1 figure supp 1-original source data/Figure 1 figure supp 1-original source data tTAU pTAU GAPDH.jpg]

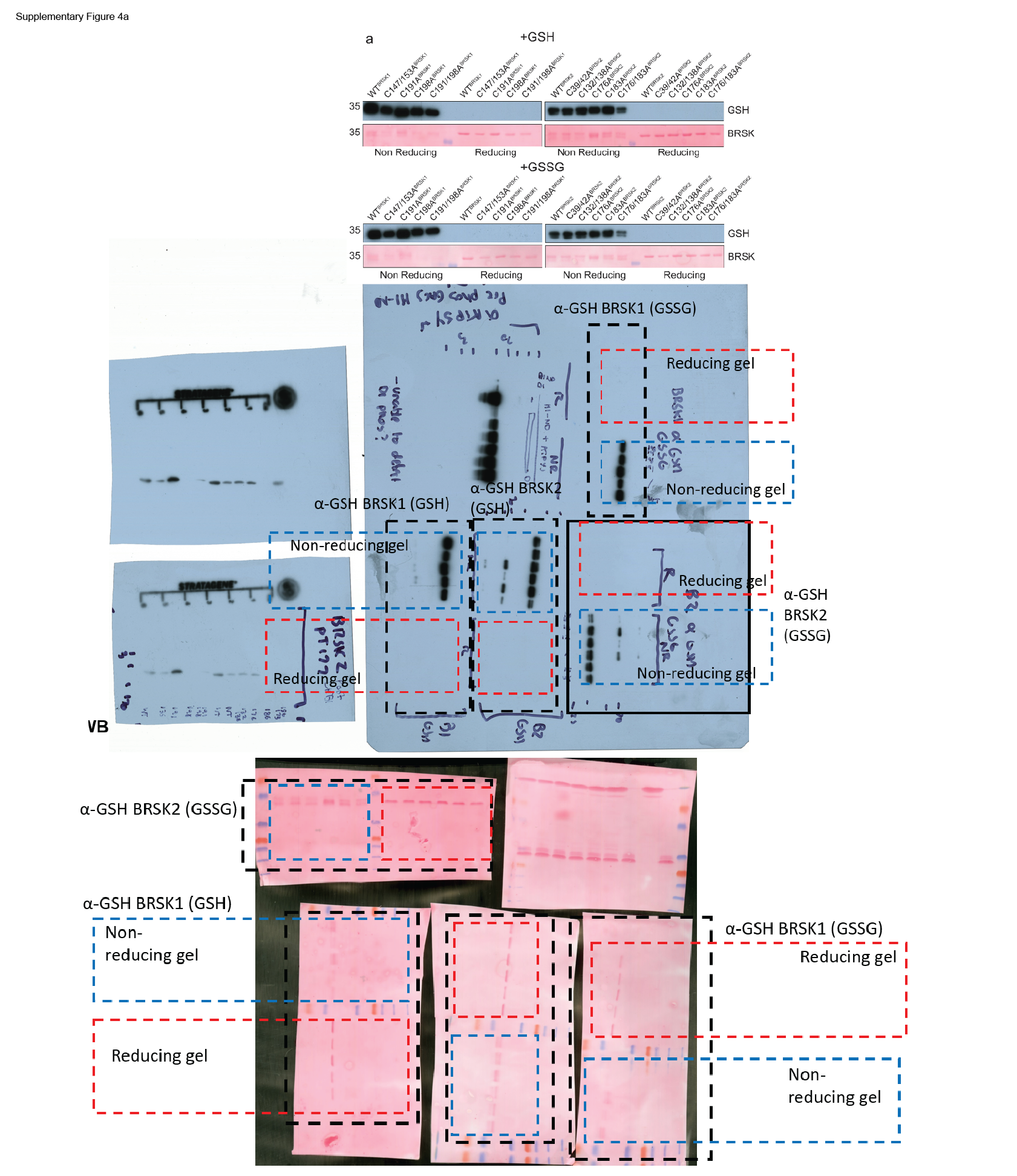

Supplement: Figure 4—figure supplement 2—source data 1. [file elife-92536-fig4-figsupp2-data1.zip › Figure 4 - figure supp 2 Source Data/Figure 4 - figure supp 2 Source Data - S4a/Figure 4 - figure supp 2 a Source Data.png]

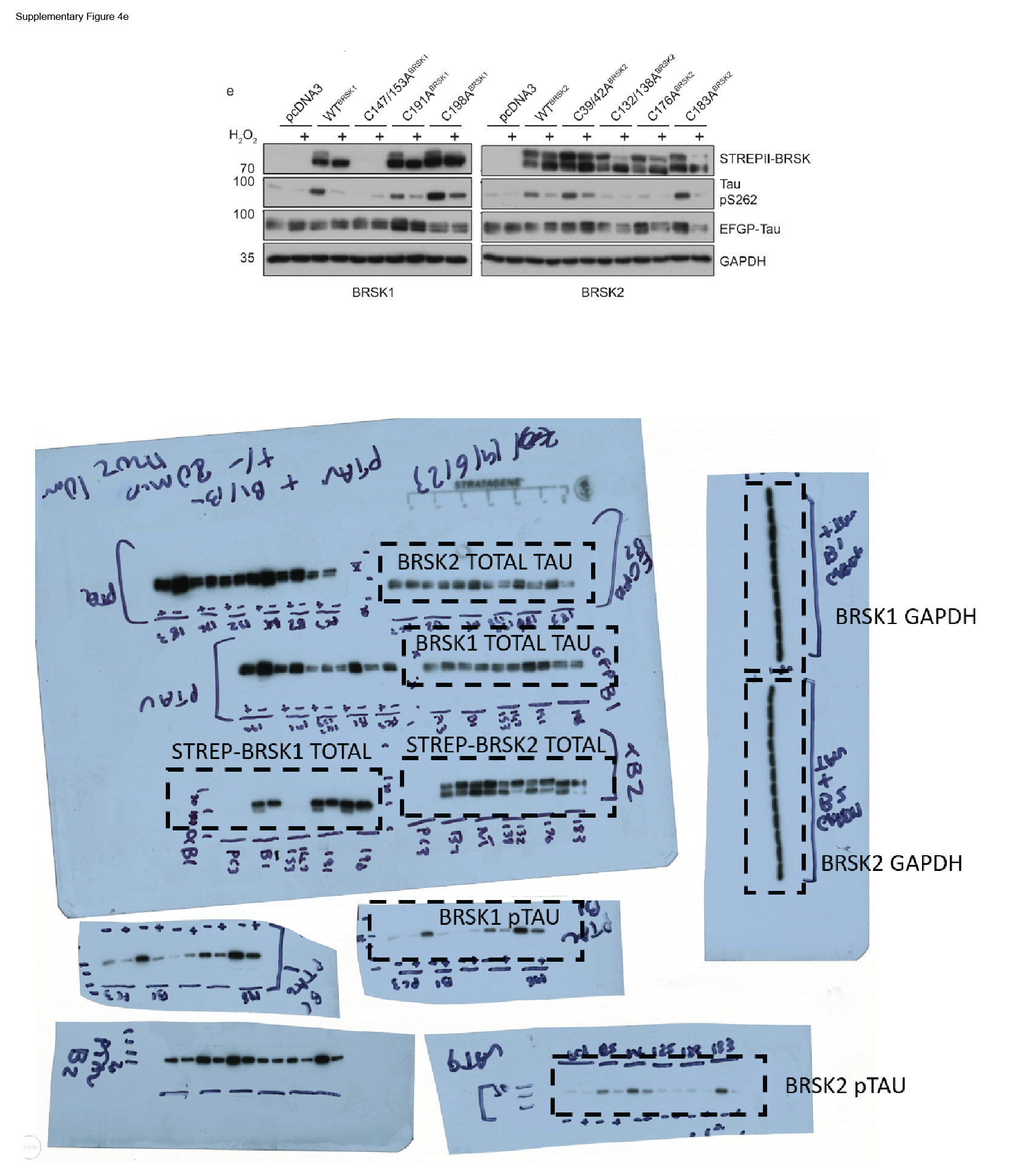

Supplement: Figure 4—figure supplement 2—source data 1. [file elife-92536-fig4-figsupp2-data1.zip › Figure 4 - figure supp 2 Source Data/Figure 4 - figure supp 2 Source Data - S4e/Figure 4 - figure supp 2e Source Data.png]

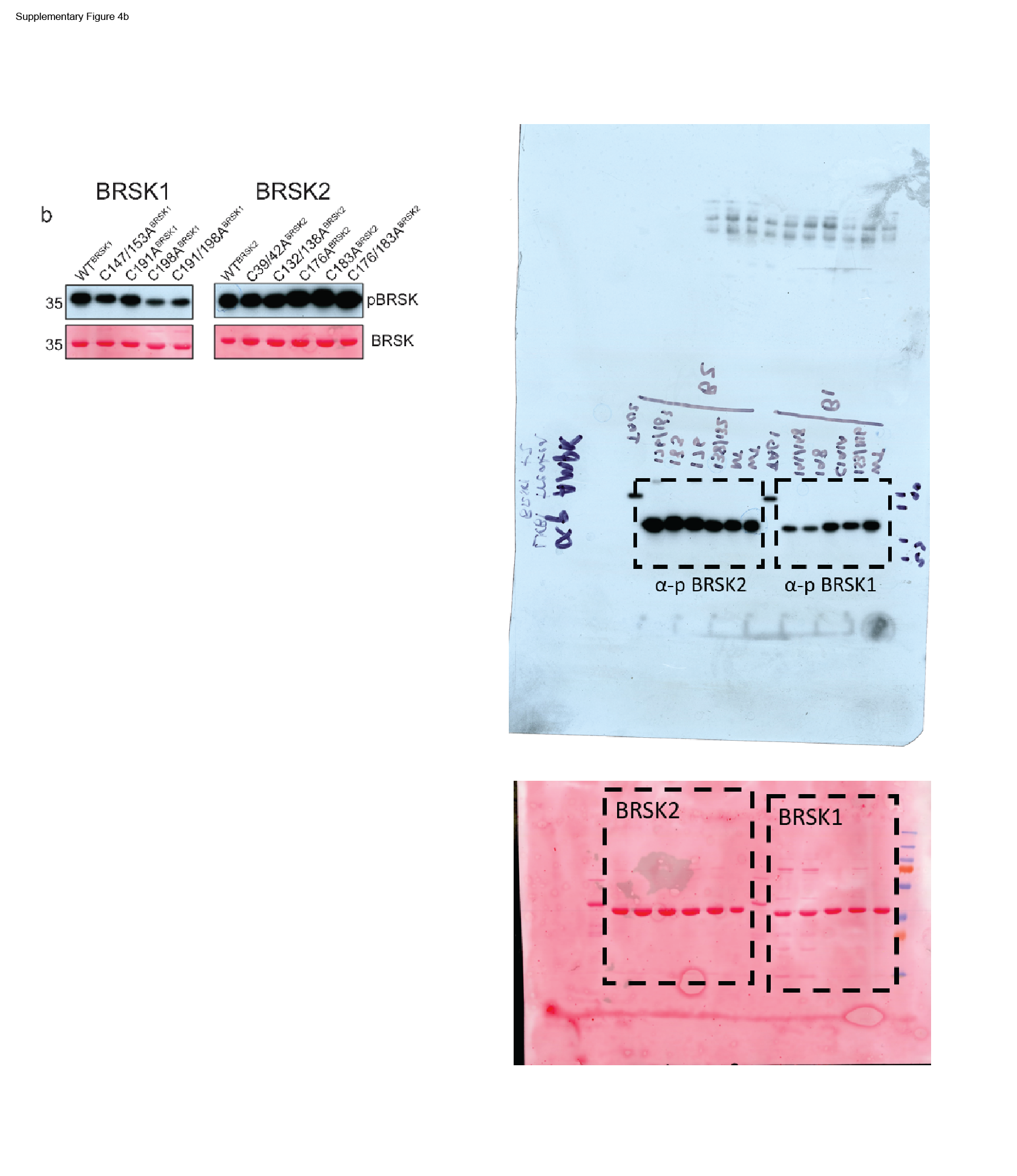

Supplement: Figure 4—figure supplement 2—source data 1. [file elife-92536-fig4-figsupp2-data1.zip › Figure 4 - figure supp 2 Source Data/Figure 4 - figure supp 2 Source Data -S4b/Figure 4 - figure supp 2 b Source Data.png]

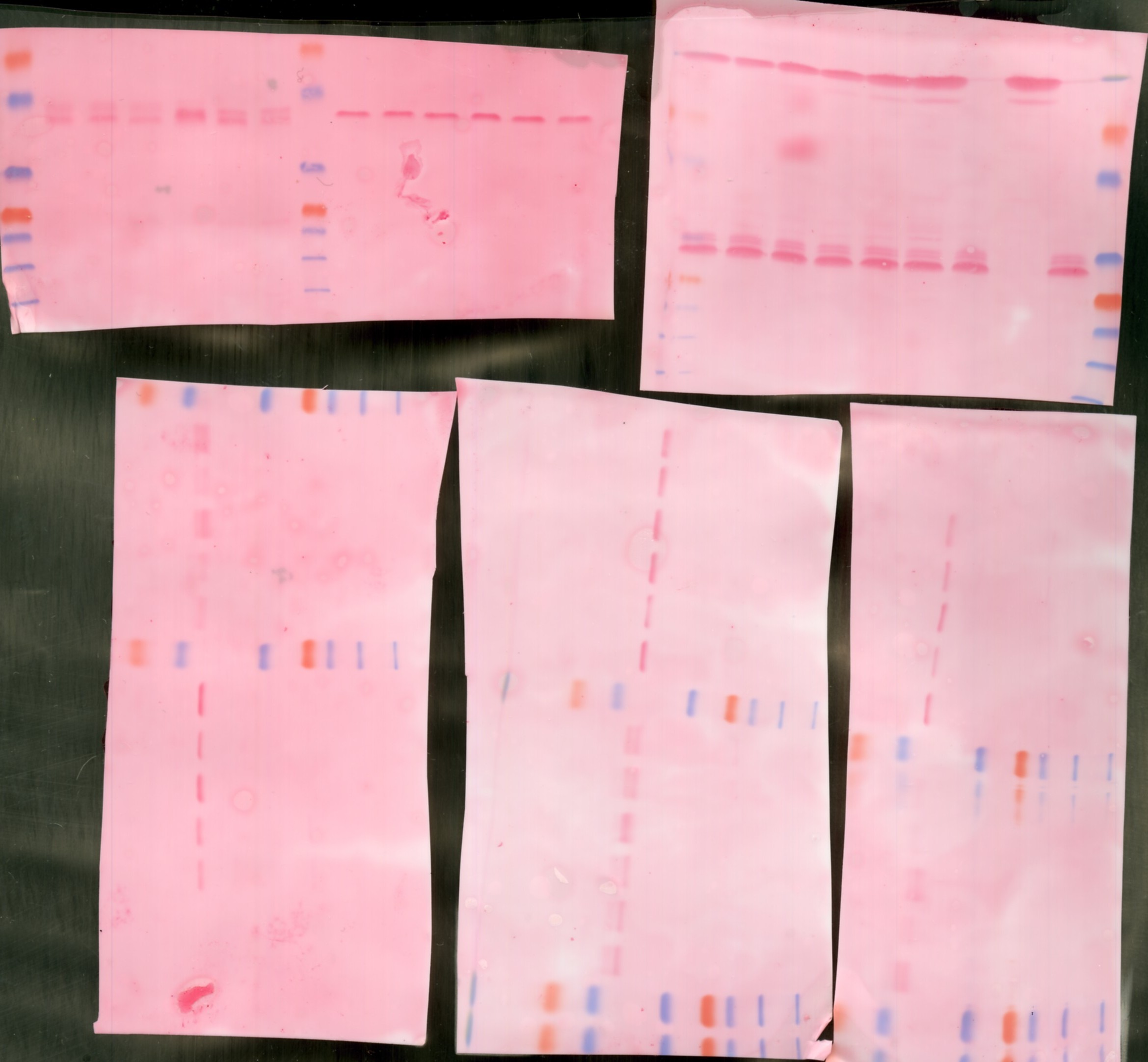

Supplement: Figure 4—figure supplement 2—source data 2. [file elife-92536-fig4-figsupp2-data2.zip › Figure 4 - figure supp 2- original source data/Figure 4 - figure supp 2- original source data - S4a/S4a PONCEAU.jpg]

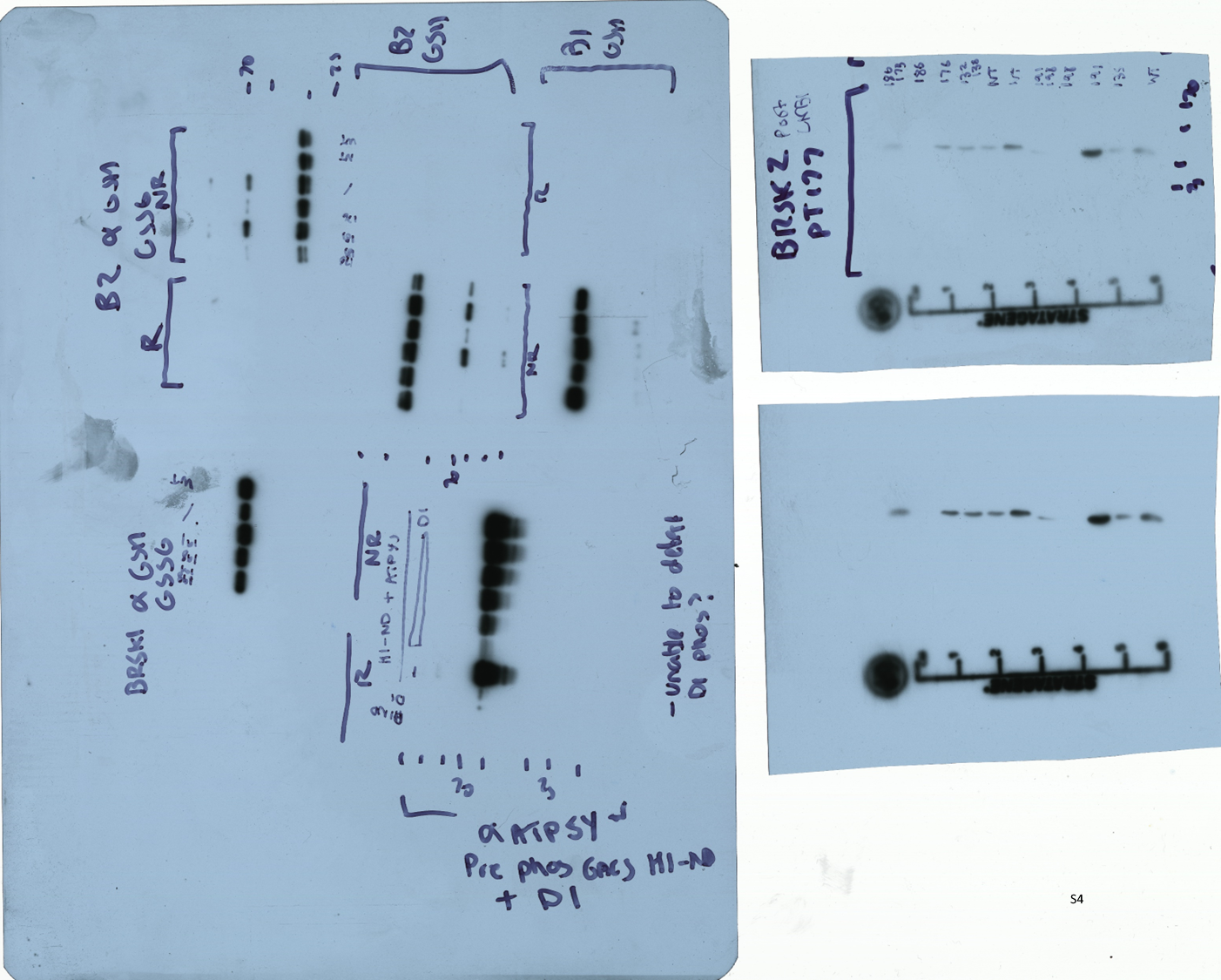

Supplement: Figure 4—figure supplement 2—source data 2. [file elife-92536-fig4-figsupp2-data2.zip › Figure 4 - figure supp 2- original source data/Figure 4 - figure supp 2- original source data - S4a/S4a Western Blot.png]

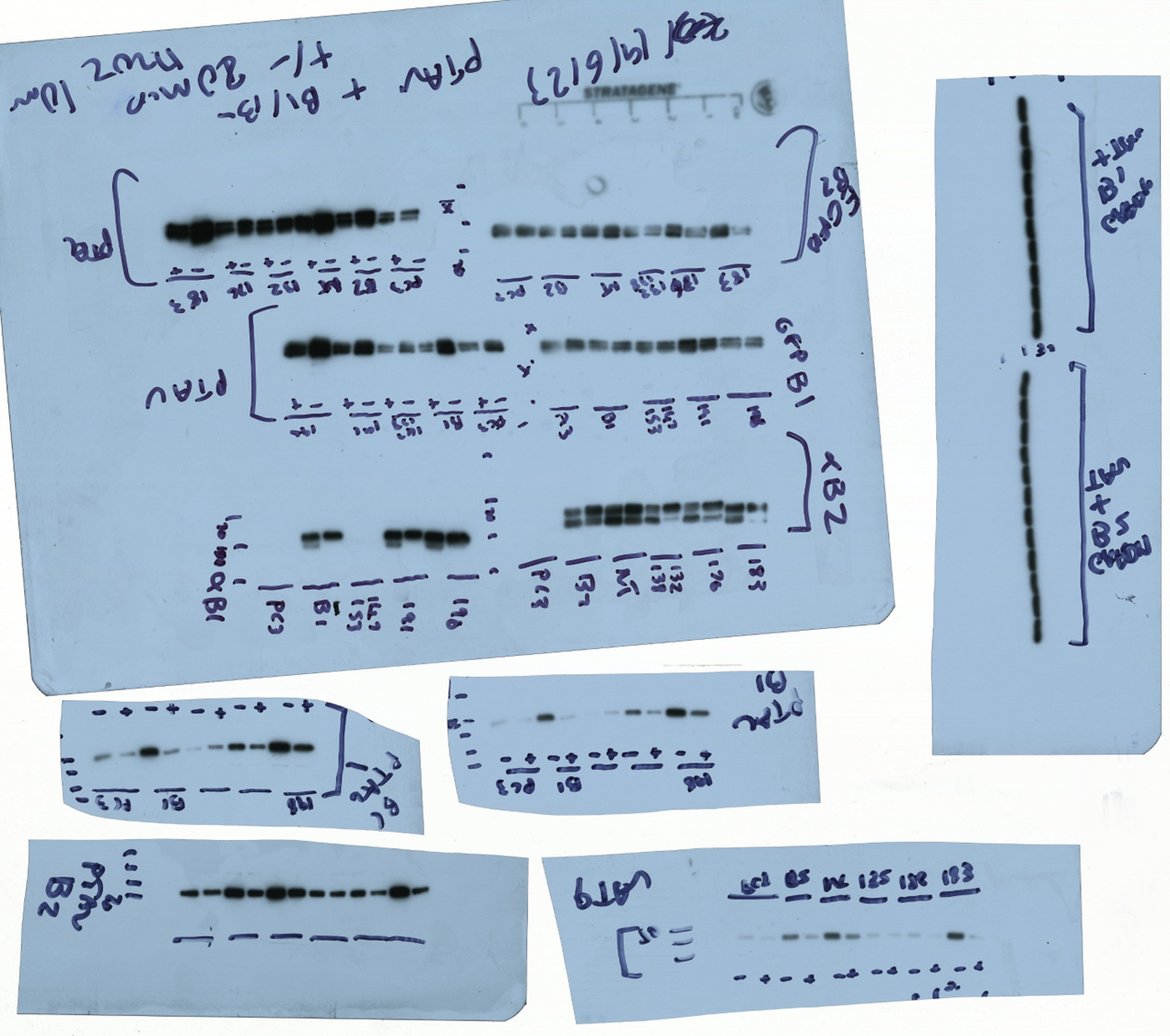

Supplement: Figure 4—figure supplement 2—source data 2. [file elife-92536-fig4-figsupp2-data2.zip › Figure 4 - figure supp 2- original source data/Figure 4 - figure supp 2- original source data - S4e/S4e.png]

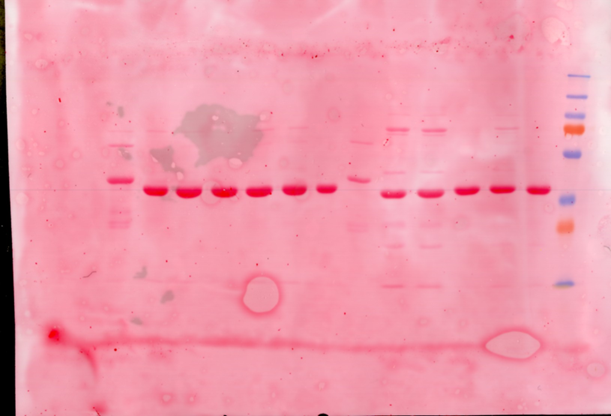

Supplement: Figure 4—figure supplement 2—source data 2. [file elife-92536-fig4-figsupp2-data2.zip › Figure 4 - figure supp 2- original source data/Figure 4 - figure supp 2- original source data -S4b/S4b PONCEAU.png]

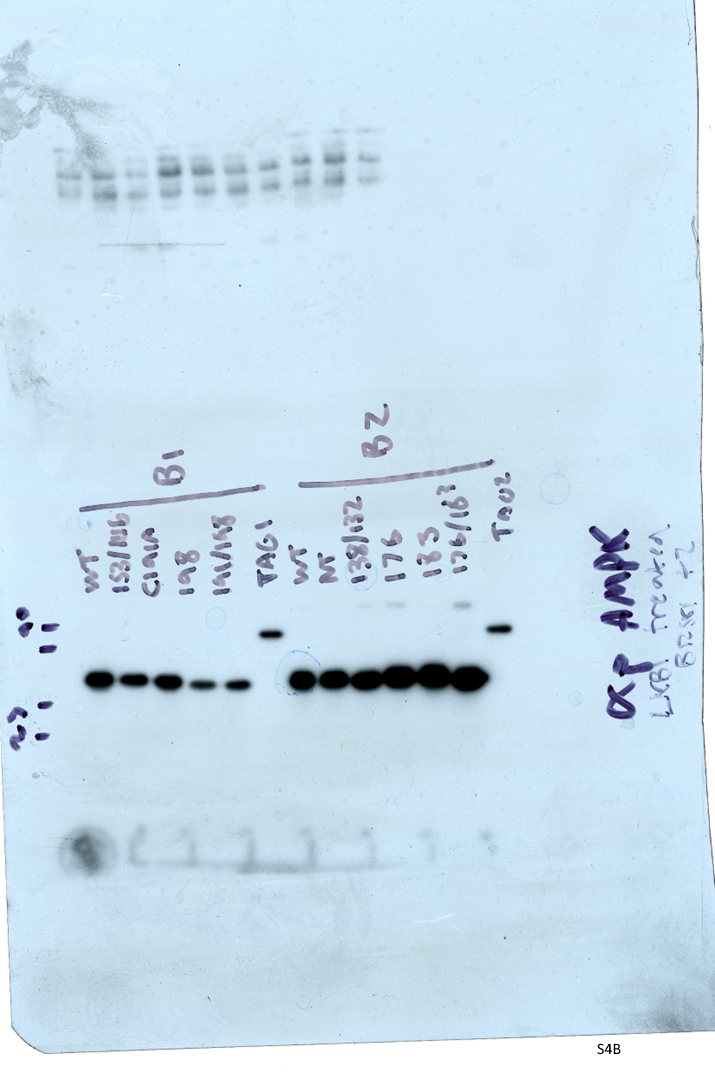

Supplement: Figure 4—figure supplement 2—source data 2. [file elife-92536-fig4-figsupp2-data2.zip › Figure 4 - figure supp 2- original source data/Figure 4 - figure supp 2- original source data -S4b/S4b Western Blot.png]

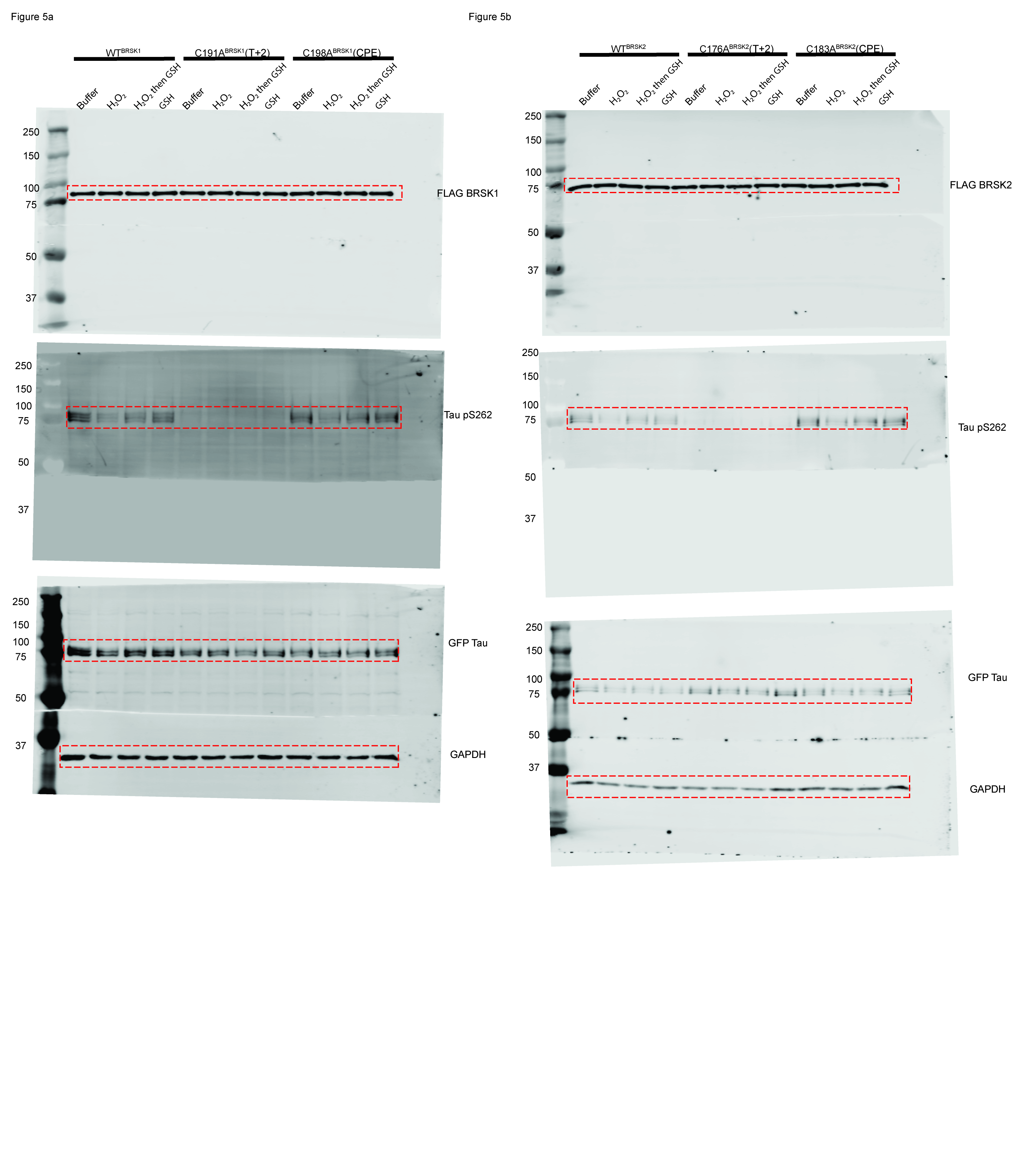

Supplement: Figure 5—source data 1. [file elife-92536-fig5-data1.zip › Figure 5-source data/Figure 5 - source data.tif]

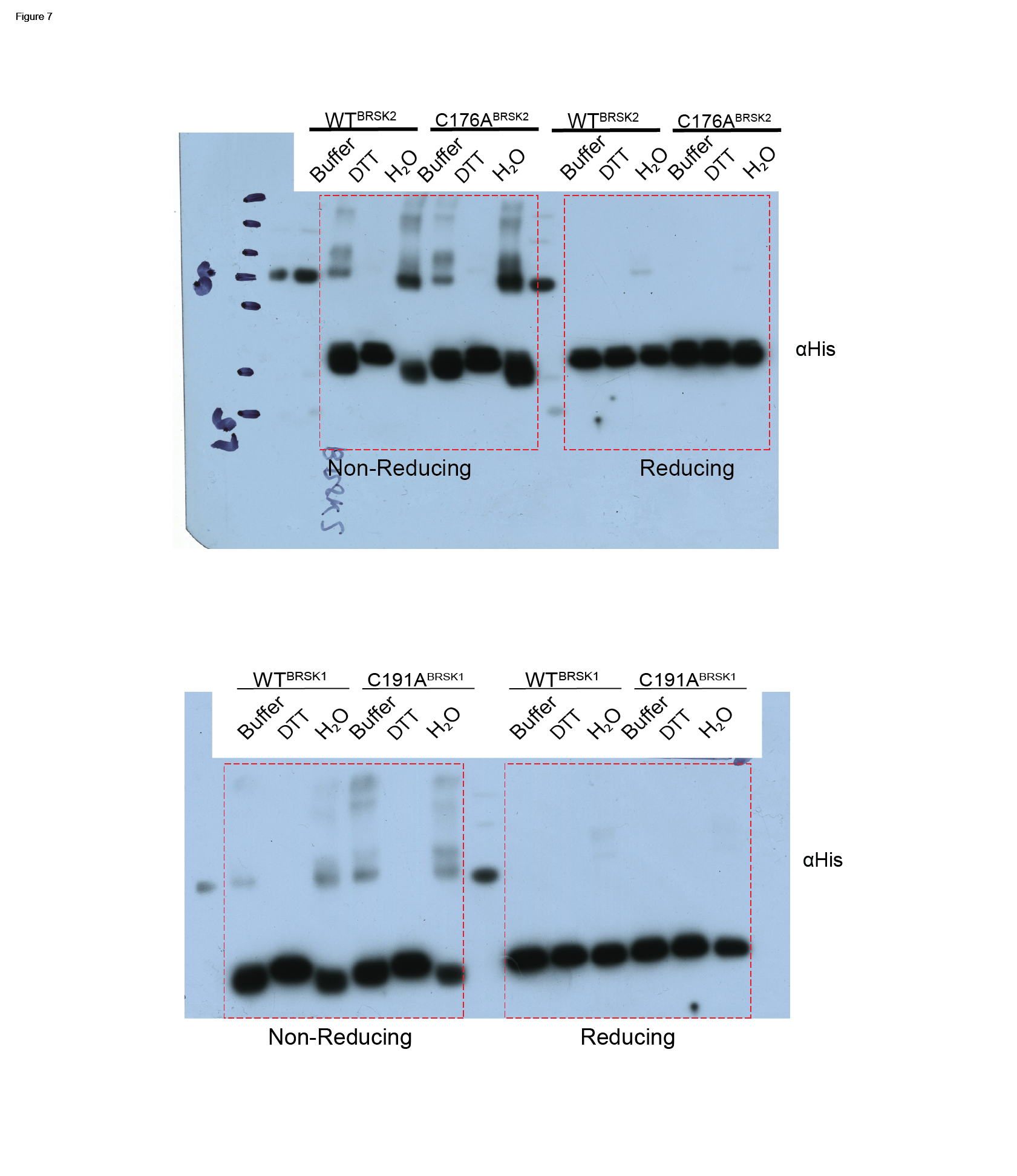

Supplement: Figure 7—source data 1. [file elife-92536-fig7-data1.zip › Figure 7-source data/Figure 7 source data.png]

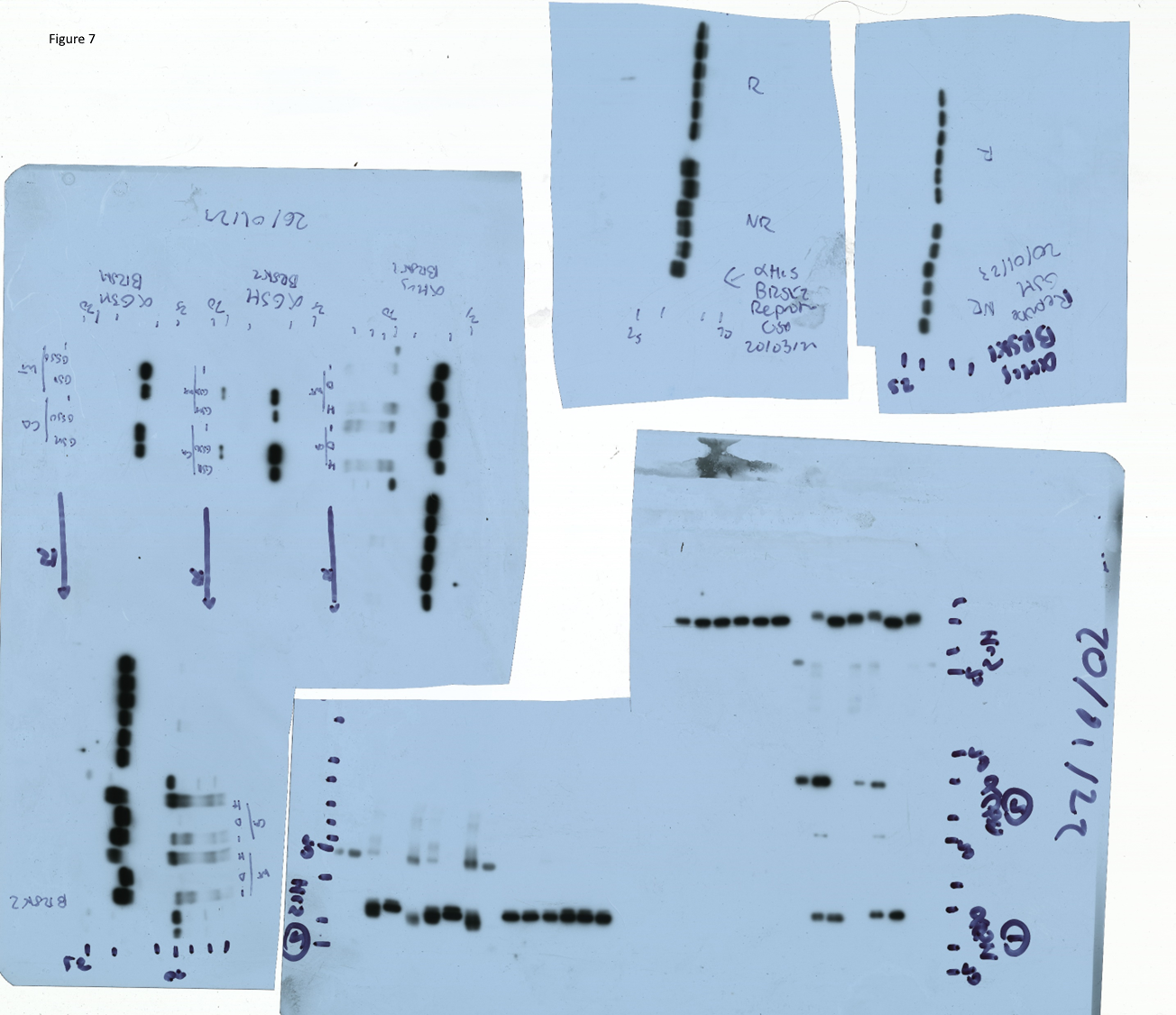

Supplement: Figure 7—source data 2. [file elife-92536-fig7-data2.zip › Figure 7-original source data/Figure 7 Source Data.png]

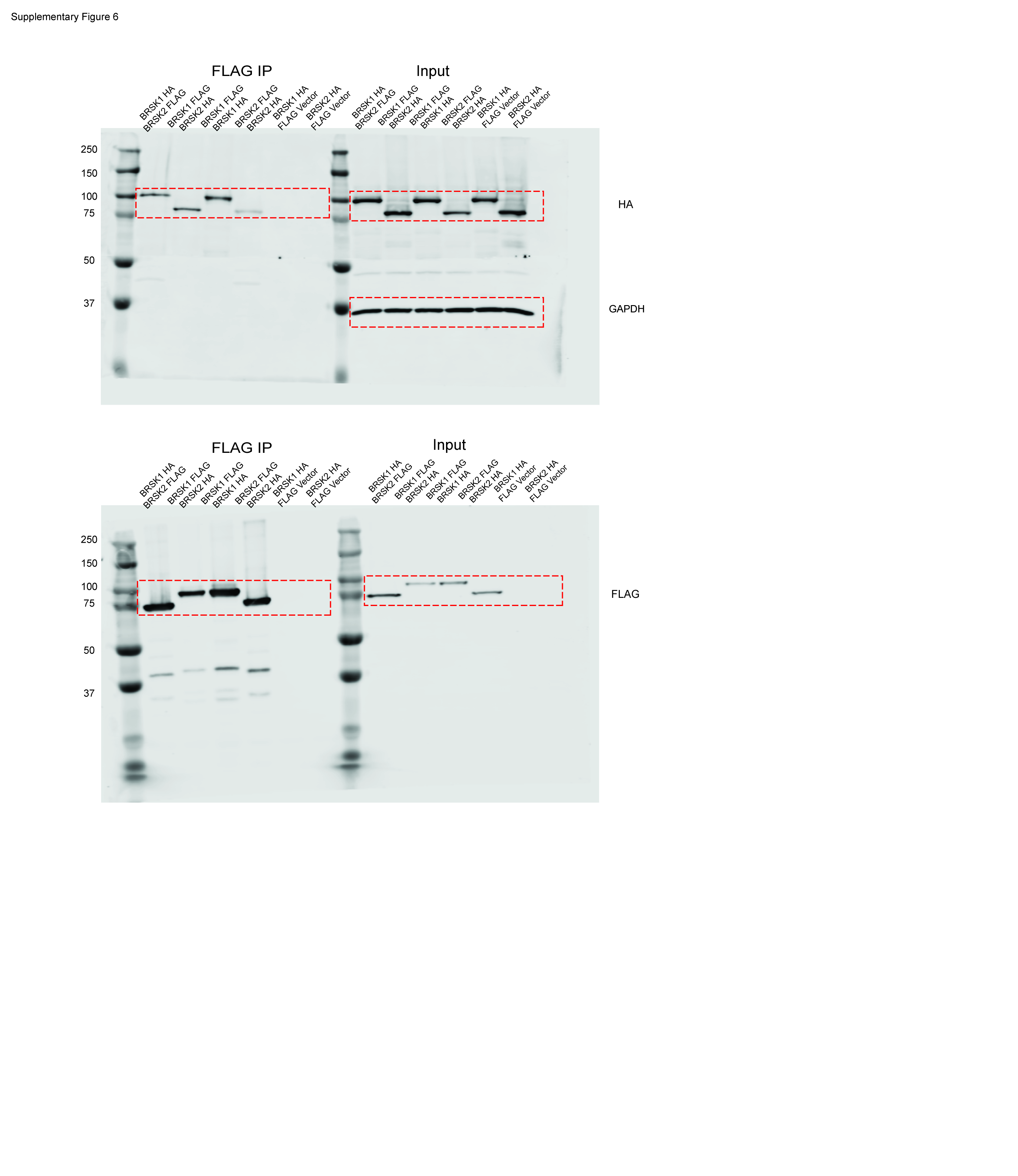

Supplement: Figure 7—figure supplement 1—source data 1. [file elife-92536-fig7-figsupp1-data1.zip › Figure 7 - figure supp 1-source data/Figure 7 - figure supp 1-source data.tif]

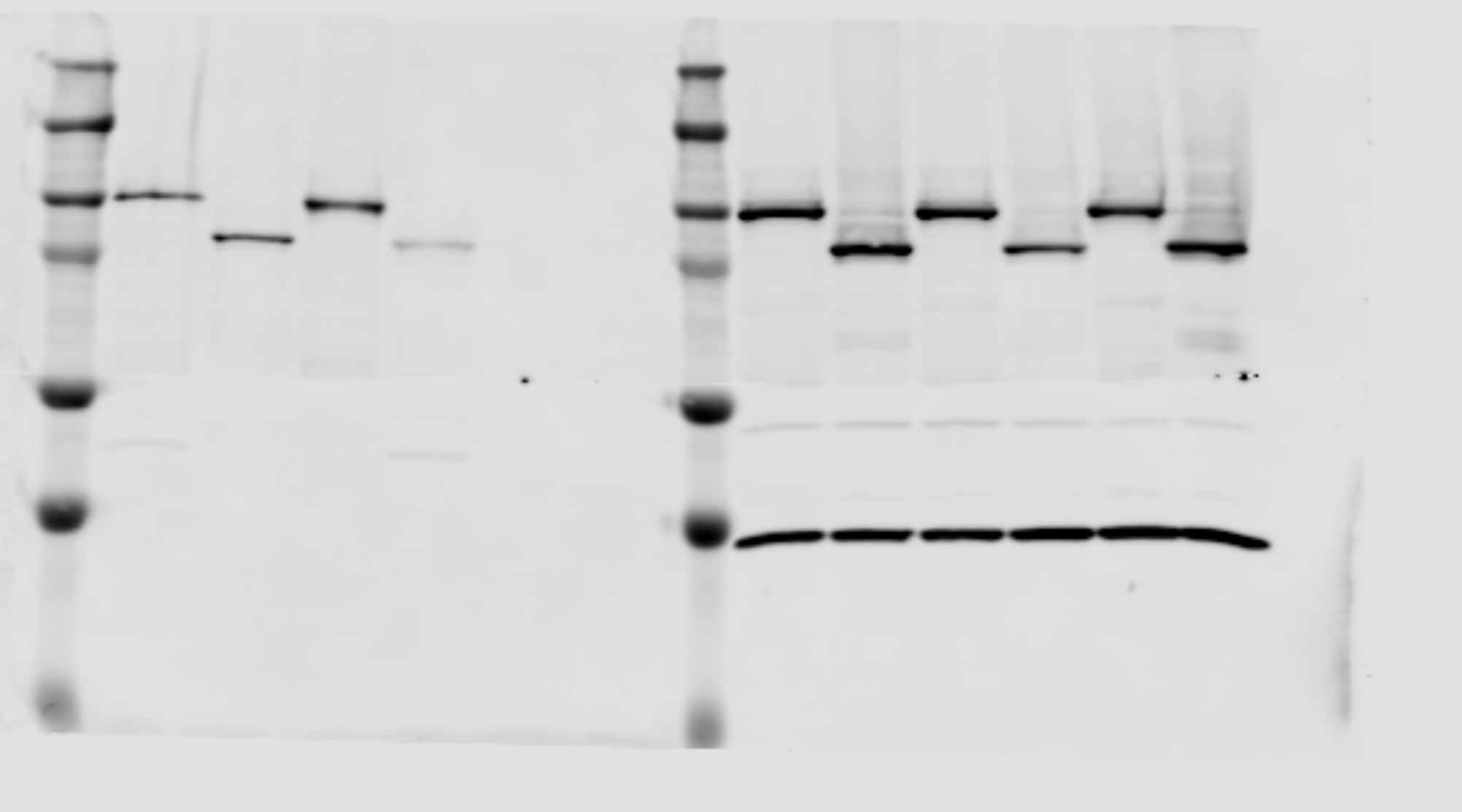

Supplement: Figure 7—figure supplement 1—source data 2. [file elife-92536-fig7-figsupp1-data2.zip › Figure 7 - figure supp 1- original source data a-source data/6a - HA GAPDH.tif]
